# Electrophoresis of an Oil Drop in a Charged Polymer Gel Medium: Coupled Effects of Drop Electrohydrodynamics and Gel Electroosmosis

**DOI:** 10.3390/gels12040302

**Published:** 2026-04-01

**Authors:** Hiroyuki Ohshima

**Affiliations:** Research Institute for Science and Technology, Tokyo University of Science, 2641 Yamazaki, Noda 278-8510, Chiba, Japan; ohshima@rs.noda.tus.ac.jp

**Keywords:** gel electrophoresis, electrophoretic mobility, oil drop, charged polymer gel medium, Marangoni effect, hydrodynamic slip

## Abstract

We develop a theoretical description of the electrophoretic migration of a weakly charged oil drop dispersed in a dilute polymer gel carrying fixed charges and saturated with an aqueous electrolyte solution. In contrast to neutral gels, a charged polymer network generates electroosmotic flow under an applied electric field, which couples with the electrohydrodynamic motion of the drop. The observed electrophoretic velocity therefore reflects the combined effects of drop-induced flow and gel-driven electroosmosis. On the basis of the Baygents–Saville theory, the drop surface charge is assumed to originate from specific ion adsorption at the oil–water interface, while no mobile ions are present inside the drop. Working within the Brinkman–Debye–Bueche porous-medium model for the gel and employing a linearized treatment valid for low zeta potential, we obtain a simple analytical expression for the electrophoretic mobility. The formulation consistently incorporates Marangoni stresses arising from spatial variations in interfacial tension, and hydrodynamic slip at the oil–water interface, which can be significant for hydrophobic drops in aqueous media. The resulting mobility expression explicitly separates the contribution associated with the intrinsic electrohydrodynamic response of the drop from that due to electroosmosis of the charged gel matrix. This analytical form enables experimental mobility data to be used not only to estimate the zeta potential of the drop but also to evaluate the electroosmotic mobility of the polymer gel medium. The present theory thus provides a physically transparent and experimentally useful framework for characterizing electrokinetic transport in charged soft porous media.

## 1. Introduction

The electrophoretic behavior of dispersed particles in polymer gels differs fundamentally from that in free electrolyte solutions [1,2,3,4,5], because the gel matrix modifies both hydrodynamic transport and electrostatic interactions. Over the past decades, a wide range of theoretical analyses has been developed for various types of dispersed entities in polymer gels. These include studies on rigid spherical particles [6,7,8,9,10,11,12,13,14,15,16,17,18,19,20], biopolymers [21], soft colloidal particles [22,23,24,25,26], and liquid drops [27,28,29]. In addition, transient and time-dependent responses have been investigated in several works [30,31,32,33,34,35]. More recent studies have addressed thermophoresis in gels [36] and two-particle electrophoresis in gel media [37]. Most theoretical treatments describe the polymer matrix using the Brinkman–Debye–Bueche model [38,39].

Liquid drops, including oil drops [40,41,42,43,44,45,46,47,48,49,50,51,52,53,54,55,56,57,58], exhibit electrokinetic characteristics that differ from those of rigid particles. Internal circulation within the drop and Marangoni stresses at the interface can significantly modify the surrounding flow field, as clearly demonstrated by Baygents and Saville [42].

In our previous study [58], we derived an analytical expression for the electrophoretic mobility of weakly charged oil drops dispersed in an uncharged dilute polymer gel based on the Baygents–Saville model [42]. In that formulation, the polymer gel was treated as a porous medium that influences the motion of the drop solely through hydrodynamic resistance, without contributing directly to the electrostatic field. This approach allowed us to capture the essential feature of hydrodynamic screening arising from the presence of the polymer network, whereby the flow field generated by the moving drop is attenuated within the gel. The resulting analytical expression provided a clear description of how the permeability of the gel affects the electrophoretic mobility. However, since the gel was assumed to be electrically neutral, electrostatic interactions associated with the polymer matrix itself were not taken into account, and therefore the possible coupling between electrostatics and hydrodynamics within the gel was neglected.

In contrast, when the polymer gel carries fixed charges, the electrohydrodynamic behavior becomes qualitatively different and significantly more complex. In such systems, the applied electric field acts not only on the diffuse ionic layer surrounding the drop but also on the counterion cloud associated with the fixed charges of the polymer network. This additional electric body force acting within the gel gives rise to a bulk electroosmotic flow, which is absent in uncharged systems. The generated electroosmotic flow interacts with the electrohydrodynamic flow induced by the drop itself, leading to a nontrivial coupling between these two mechanisms. As a consequence, the observed electrophoretic mobility can no longer be interpreted solely in terms of the drop properties, but instead reflects a combined effect involving both drop electrophoresis and gel-driven electroosmosis. This coupling is expected to play a crucial role in determining the overall transport behavior in charged soft porous media.

The present work extends our previous theory [58] to the case of charged polymer gels by explicitly incorporating the effects of fixed gel charges and the resulting electroosmotic flow into the theoretical framework. In addition to this extension, interfacial physicochemical effects are also taken into account in a consistent manner. In particular, Marangoni stresses arising from non-uniform ion adsorption along the drop surface are included, as well as the possibility of hydrodynamic slip at the oil–water interface. These interfacial effects can modify the stress balance at the drop surface and thereby influence the surrounding flow field. They are especially relevant for oil droplets dispersed in aqueous electrolyte solutions, where the interface may deviate from the classical no-slip boundary condition due to its physicochemical properties.

By incorporating these elements within a unified theoretical framework, the present analysis provides a more comprehensive description of electrophoresis in charged porous media. The theory yields a simple analytical expression for the electrophoretic mobility in the low–zeta-potential limit, while still capturing the essential coupling between electrostatic, hydrodynamic, and interfacial effects. It therefore offers a physically consistent and analytically tractable basis for interpreting electrophoretic phenomena in complex soft matter systems such as charged polymer gels and related porous media.

In addition to the specific system analyzed here, the present theoretical framework can provide a basis for further extensions to more complex situations. For example, it may be adapted to describe drops possessing finite internal conductivity, non-uniformly charged gels, or systems subjected to oscillating electric fields. Although the present theory is restricted to spherical oil drops, the underlying methodology can be extended to other types of dispersed entities, including vesicles, microgels, and biological cells, by taking into account other physical effects such as deformability, heterogeneous internal conduction, or spatial variations in interfacial properties. General discussions of the electrophoresis of soft particles and related theoretical developments can be found in the review by Ashrafizadeh et al. [59].

The analytical mobility expression obtained in this study may also be relevant to practical soft-matter systems. Potential applications include electrically driven motion of drug-encapsulating oil drops through hydrogels, biomimetic separation technologies, and drop-based microfluidic devices, where precise control of drop transport in applied electric fields is required (see, e.g., Liu et al. [60]).

## 2. Results and Discussion

### 2.1. Governing Equations

The theoretical formulation presented here is based on, and closely related to, that developed in Refs. [54,58].

#### 2.1.1. Fluid Flow and Ion Transport

**Figure 1:**
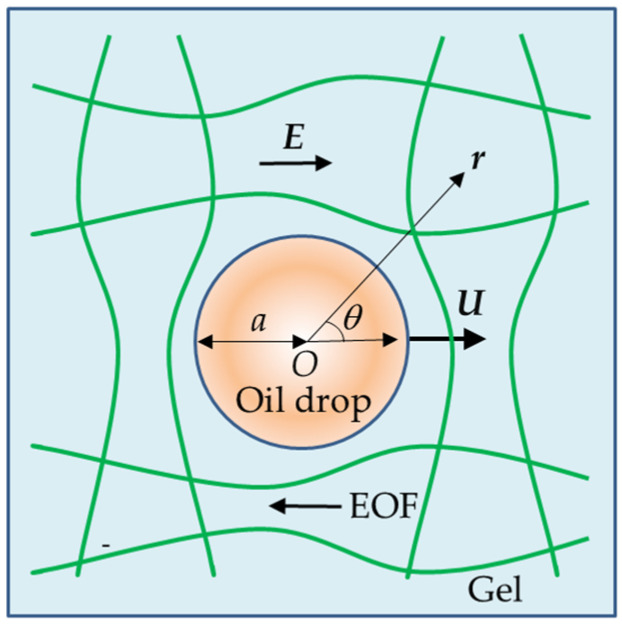
Electrophoresis of a charged spherical oil drop with velocity ***U*** in a charged polymer gel medium under an applied electric field ***E***. In a charged gel medium, an electroosmotic flow (EOF) is generated (see Equation (19)).

We consider a spherical oil drop with radius *a*, characterized by a zeta potential *ζ*, and relative permittivity *ε*_d_, translating with a constant velocity ***U*** in a charged polymer gel of effectively unbounded extent. The gel contains an electrolyte solution of viscosity *η* and relative permittivity *ε*_r_. The drop transport is induced by a uniform external electric field ***E*** (Figure 1).

The interfacial charging mechanism of the drop follows the model proposed by Baygents and Saville [43], where the drop surface charge originates from ionic adsorption. The surrounding gel is described as a Brinkman–Debye–Bueche type continuum [39,40], in which polymer segments are uniformly distributed and act as frictional obstacles to the solvent flow.

The gel phase contains fixed charges with density *ρ*_fix_, mobile electrolyte ions with number density *ρ*_e_(***r***), and gel counterions with number density *ρ*_c_(***r***). In the absence of fixed charges, both *ρ*_fix_ and *ρ*_c_(***r***) vanish. The electrolyte consists of *N* ionic species with valence *z_i_*, bulk number density $n_{i}^{\infty }$, and drag coefficient *λ_i_* (*i* = 1, 2, …, *N*). The counterions of the gel are regarded as the *N*+1-th species, having valence *z_N_*_+1_, bulk density $n_{N+1}^{\infty }$, and drag coefficient *λ_N_*_+1_. The overall electroneutrality condition reads

(1) $$\sum_{i=1}^{N+1}z_{i}{en}_{i}^{\infty }+\rho_{fix}=0$$

where *e* denotes the elementary electric charge. Here, the *N*+1-th ionic species represents the counterions associated with the fixed charges of the gel matrix, which are introduced to ensure overall electroneutrality of the system. These counterions are mobile within the gel phase and contribute to the electrostatic screening, and hence to the Debye length, in the same way as the other ionic species. It should be noted that, in real systems, when such counterions are chemically identical to electrolyte ions, they cannot be experimentally distinguished, and the present distinction is therefore conceptual, reflecting their different physical origins.

A coordinate system (*r*, *θ*, *ϕ*) is introduced with its origin fixed at the drop center, and the polar axis aligned with the applied field ***E***. Because of spherical symmetry, the velocity ***U*** of the drop is parallel to ***E***. In the absence of the external field, the drop carries a zeta potential *ζ* at *r* = *a*, where *r* = |***r***|.

The main assumptions are as follows: (i) The Reynolds numbers inside and outside the drop are sufficiently low so that inertial effects can be neglected; both fluids are regarded as incompressible. (ii) The applied external electric field ***E*** is weak, and all induced quantities are linear in ***E***. (iii) Surface tension is sufficiently large to preserve the spherical shape of the drop. (iv) No ions are dissolved inside the drop. (v) Ionic adsorption at the surface obeys a linear isotherm. (vi) Tangential slip is allowed at the interface. (vii) Ionic species cannot cross the drop surface.

The electrohydrodynamic problem is formulated in a reference frame moving with the drop, in which the origin is fixed at the center of the drop and the drop itself is at rest.

In this frame, the fluid at infinity moves with a uniform velocity equal to the negative of the drop velocity in the laboratory frame. The drop velocity in the laboratory frame is expressed as ***U*** = (*U*cos*θ*, −*U*sin*θ*, 0), while ***E*** = (*E*cos*θ*, −*E*sin*θ*, 0), where U and E denote the signed magnitudes of the velocity and electric field, respectively.

The governing equations for the velocities ***u***(***r***) = (*u_r_*(***r***), *u_θ_*(***r***), 0) and ***u***_I_(r) = (***u***_Ir_(***r***), *u*_I*θ*_(***r***), 0) of the fluid outside and inside the drop, respectively, as well as the velocity field $v_{i}\left(r\right)=(v_{ir}\left(r\right),v_{i\theta }\left(r\right),0)$ of the *i*-th ionic species (*i* = 1, 2, …, *N* + 1) are given as follows. Because of the symmetry about the polar (*θ* = 0) axis, all variables depend only on the radial coordinate *r* and the polar angle *θ* owing to axial symmetry.

(2) $$\eta \nabla \times \nabla \times u\left(r,\theta \right)+\nabla p\left(r,\theta \right)+\rho_{e}\left(r\right)\nabla \psi \left(r,\theta \right)+\gamma \left(u\left(r,\theta \right)+U\right)=0$$

(3) $$\eta_{d}\nabla \times \nabla \times u_{I}\left(r,\theta \right)+\nabla p_{I}\left(r,\theta \right)=0$$

(4) $$\nabla \cdot u\left(r,\theta \right)=0$$

(5) $$\nabla \cdot u_{I}\left(r,\theta \right)=0$$

(6) $$v_{i}\left(r,\theta \right)=u\left(r,\theta \right)-\frac{1}{\lambda_{i}}\nabla \mu_{i}\left(r,\theta \right)$$

(7) $$\mu_{i}\left(r,\theta \right)=\mu_{i0}+z_{i}e\psi \left(r,\theta \right)+kTlnn_{i}\left(r,\theta \right)$$

(8) $$\nabla \cdot \left(n_{i}\left(r,\theta \right)v_{i}\left(r,\theta \right)\right)=0$$

(9) $$∆\psi \left(r,\theta \right)=-\frac{\rho_{e}\left(r,\theta \right)}{\varepsilon_{r}\varepsilon_{0}}$$

(10) $$\rho_{e}\left(r,\theta \right)=\sum_{i=1}^{N+1}z_{i}en_{i}\left(r,\theta \right)$$

(11) $$∆\psi_{I}\left(r,\theta \right)=0$$

Here, *p*(*r*, *θ*) and *p*_I_(*r*, *θ*) represent the pressures outside and inside the drop, respectively. The charge density *ρ*_e_(*r*, *θ*) is given by Equation (10). The electric potentials outside and inside the drop are given by *ψ*(*r*, *θ*) and *ψ*_I_(*r*, *θ*), respectively. For the *i*-th ionic species at position ***r***, *μ_i_*(*r*, *θ*) and *n_i_*(*r*, *θ*) denote its electrochemical potential and number density (concentration). The term *μ_i_*_0_ is a constant part of *μ_i_*(*r*, *θ*). Boltzmann’s constant is given by *k*, the absolute temperature by *T*, and *ε*_0_ is the vacuum permittivity. Here, the subscript capital I denotes quantities inside the oil drop, namely, the fluid velocity ***u***_I_(*r*, *θ*), pressure ***p***_I_(*r*, *θ*), and the electric potential *ψ*_I_(*r*, *θ*).

Equations (2)–(5) are the Navier–Stokes equations and the continuity equation describing steady incompressible flow (assumption (i)). According to the Brinkman–Debye–Bueche model [39,40], the frictional force exerted on polymer segments in the gel medium by the liquid flow ***u***(*r*, *θ*) is expressed by the term *γ*(***u***(*r*, *θ*) +***U***) in Equation (2), where *γ* denotes the friction coefficient. Equation (6) indicates that the velocity ***v****_i_*(*r*, *θ*) of the *i*-th ionic species is caused by both ***u***(*r*, *θ*) and the gradient of *μ_i_*(*r*, *θ*), as expressed in Equation (7). The continuity equation for the *i*-th ionic species is given by Equation (8). Equation (9) denotes the Poisson equation for the region outside the drop (*r* > *a*), while Equation (11) is the Laplace equation for the region inside the drop (0 ≤ *r* < *a*).

#### 2.1.2. Ion Adsorption at the Drop Surface

Consider the ions adsorbed on the drop surface (*r* = *a*). Let $n_{i}^{s}\left(\theta \right)$, $\mu_{i}^{s}\left(\theta \right)$, $v_{i}^{s}\left(\theta \right)$, and $\lambda_{i}^{s}$ be the surface number density (concentration), electrochemical potential, velocity, and drag coefficient of the *i*-th adsorbed ions, respectively. These quantities, which are functions of *θ* only, are governed by:

(12) $$v_{i}^{s}\left(\theta \right)=u\left(a,\theta \right)-\frac{1}{\lambda_{i}^{s}}\nabla \mu_{i}^{s}\left(\theta \right)$$

(13) $$\mu_{i}^{s}\left(\theta \right)=\mu_{i0}^{s}+z_{i}e\psi \left(a,\theta \right)+kTlnn_{i}^{s}\left(\theta \right)$$

(14) $$\nabla_{s}\cdot \left(n_{i}^{s}\left(\theta \right)v_{i}^{s}\left(\theta \right)\right)+n_{i}\left(a,\theta \right)v_{ir}\left(a,\theta \right)=0$$

(15) $$n_{i}^{s}\left(\theta \right)=K_{i}n_{i}\left(a,\theta \right)$$

(16) $$\sigma \left(\theta \right)=\sum_{i=1}^{N+1}{z_{i}en}_{i}^{s}\left(\theta \right)=\sum_{i=1}^{N+1}{K_{i}z_{i}en}_{i}\left(a,\theta \right)$$

(17) $$\gamma \left(\theta \right)=\gamma_{0}-kT\sum_{i=1}^{N+1}n_{i}^{s}\left(\theta \right)=\gamma_{0}-kT\sum_{i=1}^{N+1}{K_{i}n}_{i}\left(a,\theta \right)$$

where $\mu_{i0}^{s}$ is a constant term independent of *r*. Equation (12) describes the surface velocity $v_{i}^{s}\left(\theta \right)$ of the *i*-th ionic species adsorbed on the drop surface, arising from the liquid velocity at the drop surface, ***u***(*a*, *θ*), and the tangential gradient of $\mu_{i}^{s}\left(\theta \right)$, given by Equation (13). Equation (14) denotes the surface continuity condition for the *i*-th ionic species, ∇_s_ being the surface divergence operator. Equation (15) states that adsorption of the *i*-th ionic species on the drop surface obeys a linear isotherm (assumption (iv)) with adsorption constant *K_i_*. This ion adsorption results in a non-uniform surface charge distribution *σ*(*θ*) given by Equation (16). The adsorption constant *K_i_* takes a nonzero value solely for ions that can adsorb to the drop surface, while it is zero for non-adsorbing ionic species, including those from the supporting electrolyte. The surface tension *γ*(*θ*) of the drop surface is given by Equation (17), where *γ*_0_ is the surface tension in the absence of the ion adsorption. The second term in Equation (17) corresponds to the reduction in surface tension caused by the adsorption of ionic species onto the drop surface.

#### 2.1.3. Boundary Conditions

The liquid velocities, ***u***(*r*, *θ*) and ***u***_I_(*r*, *θ*), must obey the following boundary conditions at the drop surface (*r* = *a*) and far from the drop (*r* ⟶ ∞). The normal components of ***u***(*r*, *θ*) and ***u***_I_(*r*, *θ*) must vanish at the drop surface (*r* = *a*) and ***u***(*r*, *θ*) must tend to −***U*** as *r* ⟶ ∞, viz.,

(18) $$u_{r}\left(a,\theta \right)=u_{Ir}\left(a,\theta \right)=0$$

(19) $$u\left(r,\theta \right)\to -U-\frac{\rho_{fix}}{\gamma }E\;as\;r\to \infty$$

Here the term (–*ρ*_fix_/*γ*)***E*** in Equation (19) corresponds to the electroosmotic flow (EOF) far from the drop due to the fixed charges of density *ρ*_fix_.

In addition, the net force acting on the drop, i.e., the sum of the hydrodynamic force ***F***_H_ and the electric force ***F***_E_, must vanish in the stationary state. These forces are obtained by integrating the hydrodynamic stress tensor ***σ***^H^(*r*, *θ*) and the Maxwell stress tensor ***σ***^M^(*r*, *θ*), respectively, over the drop surface. Therefore, we have

(20) $$F_{H}+F_{E}=0$$

The forces ***F***_H_ and ***F***_E_ are given by

(21) $$F_{H}=\int_{0}^{\pi }\left[\sigma_{rr}^{H}(r,\theta )cos\theta -\sigma_{r\theta }^{H}(r,\theta )sin\theta \right]dSE/E$$

(22) $$F_{E}=\int_{0}^{\pi }\left[\sigma_{rr}^{M}(r,\theta )cos\theta -\sigma_{r\theta }^{M}(r,\theta )sin\theta \right]dSE/E$$

where *dS* = 2*πa*^2^sin*θdθ*. The components of the hydrodynamic stress tensor ***σ***^H^(*r*, *θ*) are given by

(23) $$\sigma_{rr}^{H}(r,\theta )=-p+2\eta \frac{\partial u_{r}}{\partial r}$$

(24) $$\sigma_{r\theta }^{H}(r,\theta )=\eta \left(\frac{1}{r}\frac{\partial u_{r}}{\partial \theta }+\frac{\partial u_{\theta }}{\partial r}-\frac{u_{\theta }}{r}\right)$$

and the components of the Maxwell stress tensor ***σ***^M^(*r*, *θ*) are

(25) $$\sigma_{rr}^{M}\left(r,\theta \right)=\frac{1}{2}\varepsilon_{r}\varepsilon_{0}\left\{{\left(\frac{\partial \psi }{\partial r}\right)}^{2}-{\left(\frac{1}{r}\frac{\partial \psi }{\partial \theta }\right)}^{2}\right\}$$

(26) $$\sigma_{r\theta }^{M}(r,\theta )=\varepsilon_{r}\varepsilon_{0}\frac{\partial \psi }{\partial r}\left(\frac{1}{r}\frac{\partial \psi }{\partial \theta }\right)$$

Finally, the boundary condition for the ionic flow ***v****_i_*(*r*, *θ*) is given by

(27) $$v_{ir}\left(r,\theta \right)=0\;at\;r=a\;(i=1,2,\ldots ,N+1)$$

which follows from assumption (vii) that no electrolyte ions can penetrate the drop surface.

The product of the sliding friction coefficient *β* and the difference between the tangential velocity components *u_θ_*(*r*, *θ*) − *u*_I*θ*_(*r*, *θ*) across the drop surface (*r* = *a*) must be equal to the tangential component $\sigma_{r\theta }^{H}(a,\theta )$ of the hydrodynamic stress ***σ***^H^(*r*, *θ*):

(28) $$\beta {\left\{u_{\theta }\left(a,\theta \right)-u_{I\theta }(a,\theta )\right\}=\sigma }_{r\theta }^{H}(a,\theta )$$

Using Equation (24), Equation (28) can be rewritten as

(29) $$u_{\theta }(a,\theta )-u_{I\theta }(a,\theta )=\Lambda r\frac{\partial }{\partial r}{\left.\frac{u_{\theta }}{r}\right|}_{r=a}$$

where

(30) $$\Lambda =\frac{\eta }{\beta }$$

is the slip length, which characterizes the hydrophobicity of the drop surface. Note that Λ = 0 (or *β* = ∞) corresponds to a no-slip surface (hydrophilic surface), Λ ≠ 0 (*β* ≠ 0) to a hydrophobic surface, and Λ = ∞ (or *β* = 0) to a completely hydrophobic surface.

The tangential stress balance at the drop surface is given by

(31) $$\left\{\sigma_{r\theta }^{H}\left(a,\theta \right)+\sigma_{r\theta }^{M}\left(a,\theta \right)\right\}-\left\{\sigma_{Ir\theta }^{H}\left(a,\theta \right)+\sigma_{Ir\theta }^{M}\left(a,\theta \right)\right\}+\frac{1}{a}\frac{d\gamma (\theta )}{d\theta }=0$$

Here $\sigma_{Ir\theta }^{H}(r,\theta )$ and $\sigma_{Ir\theta }^{M}(r,\theta )$ respectively, represent the tangential components of hydrodynamic and Maxwell stresses inside the drop.

(32) $$\sigma_{Ir\theta }^{H}(r,\theta )=\eta_{d}\left(\frac{1}{r}\frac{\partial u_{Ir}}{\partial \theta }+\frac{\partial u_{I\theta }}{\partial r}-\frac{u_{I\theta }}{r}\right)$$

(33) $$\sigma_{Ir\theta }^{M}(r,\theta )=\varepsilon_{d}\varepsilon_{0}\frac{\partial \psi_{I}}{\partial r}\left(\frac{1}{r}\frac{\partial \psi_{I}}{\partial \theta }\right)$$

When the Marangoni stress is absent, Equation (31) reduces to the usual continuity condition for the tangential component of the total stress (i.e., the sum of the hydrodynamic and Maxwell stresses) across the drop surface.

The electric potentials *ψ*(*r*, *θ*) and *ψ*_I_(*r*, *θ*) together with the concentration *n_i_*(*r*, *θ*) of the *i*-th ionic species must satisfy the following boundary conditions:

(34) $$\psi_{I}\left(a,\theta \right)=\psi \left(a,\theta \right)$$

(35) $${\left.\varepsilon_{d}\frac{\partial \psi_{I}\left(r,\theta \right)}{\partial r}\right|}_{r=a^{-}}-{\left.\varepsilon_{r}\frac{\partial \psi \left(r,\theta \right)}{\partial r}\right|}_{r=a^{+}}=\frac{\sigma \left(\theta \right)}{\varepsilon_{0}}=\frac{1}{\varepsilon_{0}}\sum_{i=1}^{N+1}{K_{i}z_{i}en}_{i}\left(a,\theta \right)$$

Because the disturbances in *ψ*(*r*, *θ*) and in *n_i_*(*r*, *θ*) arising from the presence of the drop become negligible at sufficiently large distances from the drop, we obtain

(36) $$\psi \left(r,\theta \right)\to -Ercos\theta \;\;as\;r\;\to \infty$$

(37) $$n_{i}\left(r,\theta \right)\to n_{i}^{\infty }\;\;as\;r\;\to \infty$$

#### 2.1.4. Equilibrium Distributions

If the equilibrium ionic concentration *n_i_*^(0)^(*r*) follows the Boltzmann distribution, the equilibrium charge density *ρ*_e_^(0)^(*r*), and equilibrium electric potential *ψ*^(0)^(*r*) satisfy

(38) $$n_{i}^{\left(0\right)}\left(r\right)=n_{i}^{\infty }exp\left(-\frac{z_{i}e\psi^{\left(0\right)}\left(r\right)}{kT}\right)$$

(39) $$∆\psi^{\left(0\right)}\left(r\right)=-\frac{\rho_{e}^{\left(0\right)}\left(r\right)}{\varepsilon_{r}\varepsilon_{0}}$$

(40) $$\rho_{e}^{\left(0\right)}\left(r\right)=\sum_{i=1}^{N+1}z_{i}en_{i}^{\left(0\right)}\left(r\right)=\sum_{i=1}^{N+1}z_{i}en_{i}^{\infty }exp\left(-\frac{z_{i}e\psi^{\left(0\right)}\left(r\right)}{kT}\right)$$

Combining Equations (38)–(40), we obtain the following Poisson-Boltzmann equation:

(41) $$∆\psi^{\left(0\right)}\left(r\right)=-\frac{1}{\varepsilon_{r}\varepsilon_{0}}\sum_{i=1}^{N+1}z_{i}en_{i}^{\infty }exp\left(-\frac{z_{i}e\psi^{\left(0\right)}\left(r\right)}{kT}\right)$$

The boundary conditions for *ψ*^(0)^(*r*) are $\psi^{\left(0\right)}\left(a\right)=\zeta$ and $\psi^{\left(0\right)}\left(r\right)\to 0\;as\;r\;\to \infty .$

The equilibrium number density (concentration) of the *i*-th ionic species adsorbed onto the drop surface is denoted by $n_{i}^{s,(0)}$ and is given by

(42) $$n_{i}^{s,(0)}=K_{i}n_{i}^{\left(0\right)}\left(a\right)=K_{i}n_{i}^{\infty }exp\left(-\frac{z_{i}e\zeta }{kT}\right)$$

By use of Equation (42), the equilibrium surface charge density, denoted by *σ*^(0)^, can be written as

(43) $$\sigma^{\left(0\right)}=\sum_{i=1}^{N+1}{z_{i}en}_{i}^{s,(0)}=\sum_{i=1}^{N+1}K_{i}z_{i}en_{i}^{\left(0\right)}\left(a\right)=\sum_{i=1}^{N+1}K_{i}z_{i}en_{i}^{\infty }exp\left(-\frac{z_{i}e\zeta }{kT}\right)$$

Here, $\psi_{I}^{\left(0\right)}$ and $n_{i}^{s,\left(0\right)}$ are constants independent of *r*. This is because, in the absence of the electric field ***E***, there are no electrolyte ions inside the drop, and the adsorbed ions are uniformly distributed over the drop surface. Under these conditions, the electric potential inside the drop, which is obtained as the solution to the Laplace equation (Equation (11)), becomes constant throughout the interior. We thus obtain

(44) $${\left.\frac{d\psi^{\left(0\right)}(r)}{dr}\right|}_{r=a^{+}}=-\frac{\sigma^{\left(0\right)}}{\varepsilon_{r}\varepsilon_{0}}=-\frac{1}{\varepsilon_{r}\varepsilon_{0}}\sum_{i=1}^{N+1}K_{i}z_{i}en_{i}^{\infty }exp\left(-\frac{z_{i}e\zeta }{kT}\right)$$

For the case of low potentials, Equation (41) may be linearized as

(45) $$∆\psi^{\left(0\right)}\left(r\right)=\kappa^{2}\psi^{\left(0\right)}\left(r\right)$$

where

(46) $$\kappa ={\left(\frac{\sum_{i=1}^{N+1}z_{i}^{2}e^{2}n_{i}^{\infty }}{\varepsilon_{r}\varepsilon_{0}kT}\right)}^{1/2}$$

is the Debye-Hückel parameter, and 1/*κ* represents the Debye length. Solving Equation (45) gives

(47) $$\psi^{\left(0\right)}\left(r\right)=\zeta \frac{a}{r}e^{-\kappa (r-a)}$$

with

(48) $$\zeta =\frac{a\sigma^{\left(0\right)}}{\varepsilon_{r}\varepsilon_{0}(1+\kappa a)}=\frac{a}{\varepsilon_{r}\varepsilon_{0}(1+\kappa a)}\sum_{i=1}^{N+1}K_{i}z_{i}en_{i}^{\infty }$$

(49) $$\sigma^{\left(0\right)}=\sum_{i=1}^{N+1}K_{i}z_{i}en_{i}^{\infty }$$

Equations (47)–(49) describe the potential distribution *ψ*^(0)^(*r*) around a weakly charged oil drop, together with its zeta potential *ζ* and the equilibrium surface charged density *σ*^(0)^, respectively.

### 2.2. Weak Electric Field Approximation

For a weak applied electric field ***E***, the deviations *δn_i_*(*r*, *θ*), $\delta n_{i}^{s}\left(\theta \right)$, *δψ*(*r*, *θ*), *δψ*_I_(*r*, *θ*), *δρ*_e_(*r*, *θ*), *δμ_i_*(*r*, *θ*), $\delta \mu_{i}^{s}\left(\theta \right)$, and *δσ*(*θ*) of *n_i_*(*r*, *θ*), $n_{i}^{s}\left(\theta \right)$, *ψ*(*r*, *θ*), *ψ*_I_(*r*, *θ*), *ρ*_e_(*r*, *θ*), *μ_i_*(*r*, *θ*), $\mu_{i}^{s}\left(\theta \right)$, and *σ*(*θ*) respectively, from their equilibrium values (i.e., those in the absence of ***E***) due to the applied electric field ***E*** are small. Under this condition, each quantities may be written as

(50) $$n_{i}\left(r,\theta \right)=n_{i}^{\left(0\right)}\left(r\right)+\delta n_{i}\left(r,\theta \right)$$

(51) $${n_{i}^{s}\left(\theta \right)=n}_{i}^{s,\left(0\right)}+\delta n_{i}^{s}\left(\theta \right)$$

(52) $$\psi \left(r,\theta \right)=\psi^{\left(0\right)}\left(r\right)+\delta \psi \left(r,\theta \right)$$

(53) $$\psi_{I}\left(r,\theta \right)=\psi_{I}^{\left(0\right)}+\delta \psi_{I}\left(r,\theta \right)$$

(54) $$\rho_{e}\left(r,\theta \right)=\rho_{e}^{\left(0\right)}\left(r\right)+\delta \rho \left(r,\theta \right)$$

(55) $$\mu_{i}\left(r,\theta \right)=\mu_{i}^{\left(0\right)}+\delta \mu_{i}\left(r,\theta \right)$$

(56) $$\mu_{i}^{s}\left(\theta \right)=\mu_{i}^{s,\left(0\right)}+\delta \mu_{i}^{s}\left(\theta \right)$$

(57) $$\sigma \left(\theta \right)=\sigma^{\left(0\right)}+\delta \sigma \left(\theta \right)$$

Here quantitates with superscript (0) denote the equilibrium values, i.e., in the absence of ***E***. By substituting Equations (51)–(56) into Equations (7), (9), (10), (11), (13), and (15), we find the following relations between the small quantities:

(58) $${\delta \mu }_{i}\left(r,\theta \right)=z_{i}e\delta \psi \left(r,\theta \right)+\frac{kT\delta n_{i}\left(r,\theta \right)}{n_{i}^{\left(0\right)}\left(r\right)}$$

(59) $$∆\delta \psi \left(r,\theta \right)=-\frac{\delta \rho_{e}\left(r,\theta \right)}{\varepsilon_{r}\varepsilon_{0}}$$

(60) $${\delta \rho }_{e}\left(r,\theta \right)=\sum_{i=1}^{N+1}z_{i}e{\delta n}_{i}\left(r,\theta \right)$$

(61) $$∆\delta \psi_{I}\left(r,\theta \right)=0$$

(62) $$\delta \mu_{i}^{s}\left(\theta \right)=z_{i}e\delta \psi \left(a,\theta \right)+\frac{kT\delta n_{i}^{s}\left(\theta \right)}{n_{i}^{s,\left(0\right)}}$$

(63) $${\delta n}_{i}^{s}\left(\theta \right)=K_{i}\delta n_{i}\left(a,\theta \right)$$

(64) $$\delta \sigma \left(\theta \right)=\sum_{i=1}^{N+1}z_{i}e{\delta n}_{i}^{s}\left(\theta \right)=\sum_{i=1}^{N+1}{K_{i}z}_{i}e{\delta n}_{i}\left(a,\theta \right)$$

A comparison between Equations (58) and (62) yields

(65) $$\delta \mu_{i}^{s}\left(\theta \right)={\delta \mu }_{i}\left(a,\theta \right)$$

By substituting Equations (52), (53), (57), and (64) into Equations (34) and (35), which are the boundary conditions for the electric potentials *ψ*(*r*, *θ*) and *ψ*_I_(*r*, *θ*) across the drop surface, we find

(66) $${\delta \psi }_{I}\left(a,\theta \right)=\delta \psi \left(a,\theta \right)$$

(67) $${\left.\varepsilon_{d}\frac{\partial {\delta \psi }_{I}\left(r,\theta \right)}{\partial r}\right|}_{r=a^{-}}-{\left.\varepsilon_{r}\frac{\partial \delta \psi \left(r,\theta \right)}{\partial r}\right|}_{r=a^{+}}=\frac{\delta \sigma \left(\theta \right)}{\varepsilon_{0}}=\frac{1}{\varepsilon_{0}}\sum_{i=1}^{N+1}z_{i}e{\delta n}_{i}^{s}\left(\theta \right)=\frac{1}{\varepsilon_{0}}\sum_{i=1}^{N+1}{K_{i}z_{i}e\delta n}_{i}\left(a,\theta \right)$$

With the help of Equation (58), Equation (67) can be transformed into

(68) $${\left.\varepsilon_{d}\frac{\partial {\delta \psi }_{I}\left(r,\theta \right)}{\partial r}\right|}_{r=a^{-}}-{\left.\varepsilon_{r}\frac{\partial \delta \psi \left(r,\theta \right)}{\partial r}\right|}_{r=a^{+}}=\frac{1}{\varepsilon_{0}kT}\sum_{i=1}^{N+1}{K_{i}z_{i}en}_{i}^{\infty }exp\left(-\frac{z_{i}e\zeta }{kT}\right)\left\{\delta \mu_{i}\left(a,\theta \right)-z_{i}e\delta \psi \left(a,\theta \right)\right\}$$

Similarly, by substituting Equations (17), (52), (53), (56), (63), and (65) into Equation (31), which represents the tangential stress balance on the drop surface, we obtain

$$\eta {\left.\left(\frac{1}{r}\frac{\partial u_{r}}{\partial \theta }+\frac{\partial u_{\theta }}{\partial r}-\frac{u_{\theta }}{r}\right)\right|}_{r=a^{+}}-\eta_{d}{\left.\left(\frac{1}{r}\frac{\partial u_{Ir}}{\partial \theta }+\frac{\partial u_{I\theta }}{\partial r}-\frac{u_{I\theta }}{r}\right)\right|}_{r=a^{-}}$$

(69) $$-\sum_{i=1}^{N+1}{K_{i}n^{\infty }exp\left(-\frac{z_{i}e\zeta }{kT}\right)\frac{1}{a}\frac{\partial }{\partial \theta }\delta \mu }_{i}\left(a,\theta \right)=0$$

By using Equations (6), (12), and (69), the surface continuity conditions (Equation (14)) can be rewritten as

(70) $$\frac{1}{asin\theta }\frac{\partial }{\partial \theta }\left(sin\theta u_{\theta }\left(a,\theta \right)-\frac{1}{\lambda_{i}^{s}a}\frac{\partial }{\partial \theta }\delta \mu_{i}(a,\theta )\right)-{\left.\frac{1}{\lambda_{i}a}\frac{\partial }{\partial r}\delta \mu_{i}(r,\theta )\right|}_{r=a^{+}}=0$$

Since

(71) $$\psi \left(r,\theta \right)\to -Ercos\theta ,{\;\;\psi }^{\left(0\right)}\left(r\right)\to 0,\;\;\;\delta \psi \left(r\right)\to -Ercos\theta ,{\;\;n}_{i}\left(r,\theta \right)\to n_{i}^{\infty },{\;n_{i}^{\left(0\right)}\left(r\right)\to n_{i}^{\infty },\;\;\;\delta n}_{i}\left(r,\theta \right)\to 0\;as\;r\to \infty$$

we obtain from Equation (58),

(72) $${\delta \mu }_{i}\left(r,\theta \right)\to -z_{i}eErcos\theta \;as\;r\;\to \infty \;$$

The Navier–Stokes Equations (2) and (3) can be rewritten as follows. By taking the curl of Equation (22) to eliminate the pressure term and neglecting the products of the small quantities, we finally obtain

(73) $$\eta \nabla \times \nabla \times \nabla \times u\left(r,\theta \right)+\gamma \nabla \times u\left(r,\theta \right)+\sum_{i=1}^{N+1}\nabla n_{i}^{\left(0\right)}\left(r\right)\times \nabla \delta \mu_{i}\left(r,\theta \right)=0$$

and

(74) $$\eta \nabla \times \nabla \times \nabla \times u_{I}\left(r,\theta \right)=0$$

Similarly, from Equation (8), we obtain

(75) $$\nabla \cdot \left(n_{i}^{\left(0\right)}\left(r\right)u\left(r,\theta \right)-\frac{1}{\lambda_{i}}n_{i}^{\left(0\right)}\left(r\right)\nabla \delta \mu_{i}\left(r,\theta \right)\right)=0\;$$

Now, by symmetry, the liquid velocities ***u***(*r*, *θ*) and ***u***_I(_*r*, *θ*), the deviation *δμ_i_*(*r*, *θ*) of the electrochemical potential *μ_i_*(*r*, *θ*) of the i-th ionic species, and the deviations *δψ*(*r*, *θ*) and *δψ*_Ι_(*r*, *θ*) of the electric potentials *ψ*(*r*, *θ*) and *ψ*_Ι_(*r*, *θ*), respectively, may be written as

(76) $$u\left(r,\theta \right)=\left(-\frac{2}{r}h\left(r\right)Ecos\theta ,\;\frac{1}{r}\frac{d}{dr}(rh\left(r\right))Esin\theta ,\;0\right)$$

(77) $$u_{I}\left(r,\theta \right)=\left(-\frac{2}{r}h_{I}\left(r\right)Ecos\theta ,\;\frac{1}{r}\frac{d}{dr}(rh_{I}\left(r\right))Esin\theta ,\;0\right)$$

(78) $$\delta \mu_{i}\left(r,\theta \right)=-z_{i}e\phi_{i}\left(r\right)Ecos\theta$$

(79) $$\delta \psi \left(r,\theta \right)=-Y\left(r\right)Ecos\theta$$

(80) $$\delta \psi_{I}\left(r,\theta \right)=-Y_{I}\left(r\right)Ecos\theta$$

where *h*(*r*), *ϕi*(*r*), and *Y*(*r*) are functions of *r* only. It can be demonstrated that, similarly to the case of the free-solution electrophoresis of an ion-adsorbed oil drop [55], the governing equations (Equations (2)–(11)) reduce to the following set of equations for *h_I_*(*r*), *h*(*r*), *ϕ_i_*(*r*), *Y*(*r*) and *Y*_I_(*r*):

(81) $$L(Lh-\lambda^{2}h)=G(r)$$

(82) $$L\left(Lh_{I}(r)\right)=0$$

(83) $$L\phi_{i}=g_{i}(r)$$

(84) $$LY=\frac{1}{\varepsilon_{r}\varepsilon_{o}kT}\sum_{i=1}^{N+1}z_{i}^{2}{e^{2}n}_{i}^{\left(0\right)}(r)(Y-\phi_{i})$$

(85) $$LY_{I}=0$$

with

(86) $$G\left(r\right)=-\frac{e}{\eta r}\frac{dy}{dr}\sum_{i=1}^{N+1}z_{i}^{2}n_{i}^{\infty }e^{-z_{i}y}\phi_{i}(r)$$

(87) $$g_{i}(r)=\frac{dy}{dr}\left(z_{i}\frac{d\phi_{i}}{dr}-\frac{2\lambda_{i}}{e}\frac{h}{r}\right)$$

(88) $$\lambda ={\left(\frac{\gamma }{\eta }\right)}^{1/2}$$

Here *y*(r) = *eψ*^(0)^(*r*)/*kT* represents the scaled equilibrium electric potential, *λ* is Brinkman parameter, 1/*λ* is the Brinkman screening length, and

(89) $$L=\frac{d}{dr}\frac{1}{r^{2}}\frac{d}{dr}r^{2}=\frac{d^{2}}{dr^{2}}+\frac{2}{r}\frac{d}{dr}-\frac{2}{r^{2}}$$

is a differential operator.

The boundary conditions (18), (29), (31), and (19) for ***u***(*r*, *θ*) and ***u***_I_(*r*, *θ*) reduce to

(90) $$h(a^{+})=0$$

(91) $$h_{I}(a^{-})=0$$

(92) $${\left.\frac{dh}{dr}\right|}_{r=a^{+}}-{\left.\frac{dh_{I}}{dr}\right|}_{r=a^{-}}=\Lambda {\left.\frac{d^{2}h}{dr^{2}}\right|}_{r=a^{+}}$$

(93) $$\eta_{d}{\left.\frac{d^{2}h_{I}}{dr^{2}}\right|}_{r=a^{-}}-\eta {\left.\frac{d^{2}h}{dr^{2}}\right|}_{r=a^{+}}=-\frac{1}{a}\sum_{i=1}^{N}{K_{i}z}_{i}en_{i}^{\infty }exp\left(-\frac{z_{i}e\zeta }{kT}\right)\phi_{i}(a)$$

(94) $$h\left(r\right)\to \frac{U}{2E}r+O\left(\frac{1}{r}\right)as\;r\;\to \infty$$

A solution of Equation (85) which satisfies Equation (85) is

(95) $$h_{I}\left(r\right)=A\left(r-\frac{r^{3}}{a^{2}}\right)$$

where *A* is a constant. By using Equation (95), we may combine Equations (92) and (93) to give the boundary condition involving only *h*(*r*) and *ϕ_i_*(*r*),

(96) $${\left.\frac{dh}{dr}\right|}_{r=a^{+}}-\Lambda_{eff}{\left.\frac{d^{2}h}{dr^{2}}\right|}_{r=a^{+}}=-\frac{1}{3\eta_{d}}\;\sum_{i=1}^{N}K_{i}z_{i}en_{i}^{\infty }exp\left(-\frac{z_{i}e\zeta }{kT}\right)\phi_{i}(a)$$

The boundary conditions for *δμ_i_*(*r*) (Equations (70) and (72)) can be rewritten in terms of *ϕ_i_*(*r*) as follows.

(97) $${\left.\frac{d\phi_{i}(r)}{dr}\right|}_{r=a^{+}}+2K_{i}\left\{\frac{\lambda_{i}}{z_{i}ea}{\left.\frac{dh(r)}{dr}\right|}_{r=a^{+}}-\frac{\lambda_{i}}{\lambda_{i}^{s}}\frac{\phi_{i}(a)}{a^{2}}\right\}=0$$

(98) $$\phi_{i}(r)\to r\;as\;r\;\to \infty$$

It can be shown that the force-free condition (Equation (20)) may be expressed as

(99) $${\left.\frac{d^{3}h}{dr^{3}}\right|}_{r=a^{+}}-\frac{5}{a}{\left.\frac{d^{2}h}{dr^{2}}\right|}_{r=a^{+}}-\left(\frac{18}{a^{2}}+\lambda^{2}\right){\left.\frac{dh}{dr}\right|}_{r=a^{+}}=2\int_{a}^{\infty }\left(1-\frac{3r^{3}}{2a^{3}}\right)G\left(r\right)dr-\frac{{3\rho }_{fix}\left(a\right)}{\eta }\left\{Y\left(a\right)-a\right\}$$

with

(100) $$\Lambda_{eff}=\Lambda +\frac{\eta a}{3\eta_{d}}$$

where Λ_eff_ my be regarded as an effective slip length. Note that Equation (99) corresponds to Equation (48) in our previous paper [57] for an uncharged gel medium. However, Equation (48) in Ref. [58] contains an error: the term $-\left(18/a^{2}+\lambda^{2}\right){\left.dh/dr\right|}_{r=a^{+}}$ should be added to the right-hand side of Equation (48) in Ref. [57].

The boundary conditions for *δψ*(*r*, *θ*) and *δψ*_I_(*r*, *θ*) can be expressed in terms of *Y*(*r*) and *Y*_I_(*r*) as follows.

(101) $$Y_{I}\left(a\right)=Y\left(a\right)$$

(102) $${\left.\varepsilon_{d}\frac{dY_{I}\left(r\right)}{dr}\right|}_{r=a^{-}}-{\left.\varepsilon_{r}\frac{dY\left(r\right)}{dr}\right|}_{r=a^{+}}=\frac{1}{\varepsilon_{0}kT}\sum_{i=1}^{N+1}{K_{i}z_{i}en}_{i}^{\infty }exp\left(-\frac{z_{i}e\zeta }{kT}\right)\left\{\phi_{i}\left(a\right)-z_{i}eY\left(a\right)\right\}$$

A solution of Equation (80) which satisfies Equation (73) is

(103) $$Y_{I}\left(r\right)=Br$$

where *B* is a constant. By using Equation (103), we may combine Equations (101) and (102) to give the boundary condition involving only *h*(*r*) and *ϕ_i_*(*r*),

(104) $$\varepsilon_{d}\frac{Y(a)}{a}-{\left.\varepsilon_{r}\frac{dY\left(r\right)}{dr}\right|}_{r=a^{+}}=\frac{1}{\varepsilon_{0}kT}\sum_{i=1}^{N+1}{K_{i}{z_{i}^{2}e}^{2}n}_{i}^{\infty }exp\left(-\frac{z_{i}e\zeta }{kT}\right)\left\{\phi_{i}\left(a\right)-Y\left(a\right)\right\}$$

### 2.3. General Mobility Expression

It follows from Equation (19) that the gel electrophoretic mobility *μ* of an oil drop, which is defined by ***U*** = *μ**E***, is given by

(105) $$\mu =\frac{U}{E}=\underset{r\to \infty }{\lim}\frac{2h(r)}{r}-\frac{\rho_{fix}}{\eta \lambda^{2}}$$

The second term, −*ρ*_fix_/*ηλ*^2^, represents the electroosmotic flow (EOF) through the gel phase induced by the gel counterions *ρ*_c_(***r***) of the *N*+1-th ionic species; this flow is in the same direction as the drop velocity ***U*** for *ρ*_fix_ < 0, increasing the magnitude of ***U*** relative to the case *ρ*_fix_ = 0, whereas for *ρ*_fix_ > 0 it decreases.

By solving Equation (40) subject to the boundary conditions given in Equations (46)–(48), and substituting the result into Equation (52), we obtain

(106) $$\mu =\frac{2}{3\lambda^{2}\Omega }\int_{a}^{\infty }\left[\left(1+\lambda r\right)e^{-\lambda \left(r-a\right)}-\left(1+\lambda a\right)-\frac{\lambda^{2}a^{2}}{3}\left\{\frac{a+\Lambda_{eff}\left(3+\lambda a\right)}{a+2\Lambda_{eff}}\right\}\left(1-\frac{r^{3}}{a^{3}}\right)\right]G(r)dr\;-\frac{2a\left(1+\lambda a\right)}{9\eta_{d}\left(a+2\Lambda_{eff}\right)\Omega }\;\sum_{i=1}^{N+1}K_{i}z_{i}en_{i}^{\infty }exp\left(-\frac{z_{i}e\zeta }{kT}\right)\phi_{i}\left(a\right)-\frac{\rho_{fix}}{\eta \lambda^{2}}\left[1-\frac{2\lambda a^{2}}{9\Omega }\left\{\frac{Y(a)}{a}-1\right\}\left\{\frac{a+\Lambda_{eff}\left(3+\lambda a\right)}{a+2\Lambda_{eff}}\right\}\right]$$

with

(107) $$\Omega =1+\lambda a+\left\{\frac{a+\Lambda_{eff}\left(3+\lambda a\right)}{a+2\Lambda_{eff}}\right\}\frac{\lambda^{2}a^{2}}{9}$$

Here Ω represents the modification factor for the Stokes drag on an oil drop with a slip surface moving through a gel medium.

Similarly, by solving Equation (41) subject to the boundary conditions given in Equations (50) and (51), we obtain

(108) $$\phi_{i}\left(r\right)=r-\frac{r}{3}\int_{r}^{\infty }g_{i}\left(x\right)dx-\frac{1}{3r^{2}}\int_{a}^{r}r^{3}g_{i}\left(r\right)dr-\frac{a^{3}}{6r^{2}}\left(\frac{\lambda_{i}^{s}a-2K_{i}\lambda_{i}}{\lambda_{i}^{s}a+K_{i}\lambda_{i}}\right)\int_{a}^{\infty }g_{i}\left(r\right)dr\;+\frac{a^{3}}{2r^{2}}\left(\frac{\lambda_{i}^{s}a-2K_{i}\lambda_{i}}{\lambda_{i}^{s}a+K_{i}\lambda_{i}}\right)+\frac{a^{3}}{r^{2}}\left(\frac{K_{i}\lambda_{i}^{s}\lambda_{i}}{\lambda_{i}^{s}a+K_{i}\lambda_{i}}\right)\frac{1}{z_{i}e}{\left.\frac{dh}{dr}\right|}_{r=a^{+}}$$

By evaluating *ϕ_i_*(*r*) at *r* = *a* using Equation (68) and substituting the resulting value *ϕ_i_*(*a*) together with the value of *Y*(*a*) obtained by solving Equation (84) into Equation (66), the electrophoretic mobility *μ* of an oil drop in a charged polymer gel medium can be calculated.

Equation (106) is the required general expression for the electrophoretic mobility *μ* of a charged spherical oil drop of radius *a* carrying zeta potential *ζ* in a charged polymer gel medium.

### 2.4. Results and Discussion

#### 2.4.1. Low-Potential Approximation

Let us derive an approximate expression for the electrophoretic mobility *μ*, correct to first order in the zeta potential *ζ*. For the low-zeta-potential case, the equilibrium potential distribution *ψ*^(0)^(*r*) outside the drop is given by Equation (47). In addition, we assume that the drag coefficient $\lambda_{i}^{s}$ of ions adsorbed on the drop surface is significantly larger than that of freely moving ions in the gel medium, denoted by *λ_i_*. This assumption is justified in most practical cases.

For the low potential case, under this assumption, Equation (108) reduces to

(109) $$\phi_{i}\left(r\right)=r+\frac{a^{3}}{2r^{2}}\;$$

and Equation (86) becomes

(110) $$G\left(r\right)=\frac{\varepsilon_{r}\varepsilon_{0}\kappa^{2}a\zeta }{\eta }\left(1+\frac{a^{3}}{2r^{3}}\right)\frac{1+\kappa r}{r^{2}}e^{-\kappa (r-a)}\;$$

By solving Equation (84) subject to Equation (104), we find that for the low potential case

(111) $$Y(a)=\frac{3a}{2}\left(1-\frac{\varepsilon_{d}}{\varepsilon_{d}+\varepsilon_{r}K+\frac{1}{\varepsilon_{0}kT}\sum_{i=1}^{N+1}{K_{i}{z_{i}^{2}e}^{2}n}_{i}^{\infty }}\right)\;$$

with

(112) $$K=1+\kappa a+\frac{1}{1+\kappa a}\;$$

As seen in Equation (111), unlike the case of the free-solution electrophoresis of a rigid sphere [35] or an oil drop [36], and the electrophoresis of a rigid sphere [34] or an oil drop in an uncharged gel [37], the electrophoresis of an oil drop in a charged polymer gel depends on the relative permittivity *ε*_d_ of the drop.

We now treat the case where the relative permittivity *ε*_d_ of the drop is much smaller than that of the electrolyte solution *ε*_r_. This condition holds for most practical situations, and Equation (110) reduces to

(113) $$Y(a)=\frac{3a}{2}\;$$

By substituting Equations (109), (110), and (113) into Equation (106), we finally obtain the following expression for the gel electrophoretic mobility *μ* of the oil drop:

(114) $$\mu =\frac{2\varepsilon_{r}\varepsilon_{0}\kappa^{2}a\zeta }{3\eta \lambda^{2}\Omega }\int_{a}^{\infty }\left[\left(1+\lambda r\right)e^{-\lambda \left(r-a\right)}-\left(1+\lambda a\right)-\frac{\lambda^{2}a^{2}}{3}\left\{\frac{a+\Lambda_{eff}\left(3+\lambda a\right)}{a+2\Lambda_{eff}}\right\}\left(1-\frac{r^{3}}{a}\right)\right]\left(1+\frac{a^{3}}{2r^{3}}\right)\frac{1+\kappa r}{r^{2}}e^{-\kappa (r-a)}dr\;-\frac{\varepsilon_{r}\varepsilon_{0}\zeta a\left(1+\kappa a\right)\left(1+\lambda a\right)}{3\eta_{d}\Omega \left(a+2\Lambda_{eff}\right)}-\frac{\rho_{fix}}{\eta \lambda^{2}\Omega }\left(1+\lambda a\right)$$

Equation (114) can be rewritten in terms of the exponential integrals, that is,

(115) $$\mu =\frac{{2\varepsilon }_{r}\varepsilon_{o}\zeta }{3\eta \Omega }\left[\frac{a+\Lambda_{eff}\left(3+\lambda a\right)}{a+2\Lambda_{eff}}+\frac{\kappa \lambda a}{\kappa +\lambda }+\frac{\kappa a\Lambda_{eff}\left(1+\lambda a\right)}{a+2\Lambda_{eff}}+\frac{3\kappa^{2}}{2\lambda^{2}}\left\{1+\lambda a+\frac{\lambda^{2}a^{2}}{3}\left(\frac{a+\Lambda_{eff}\left(3+\lambda a\right)}{a+2\Lambda_{eff}}\right)\right\}e^{\kappa a}E_{5}\left(\kappa a\right)\right.\;-\frac{3\kappa^{2}}{2\lambda^{2}}e^{\left(\kappa +\lambda \right)a}\left.\left\{E_{5}\left(\left(\kappa +\lambda \right)a\right)+\lambda aE_{4}\left(\left(\kappa +\lambda \right)a\right)+\frac{\lambda^{2}a^{2}}{3}E_{3}\left(\left(\kappa +\lambda \right)a\right)\right\}\right]-\frac{\varepsilon_{r}\varepsilon_{0}\zeta a\left(1+\kappa a\right)\left(1+\lambda a\right)}{3\eta_{d}\Omega \left(a+2\Lambda_{eff}\right)}-\frac{\rho_{fix}}{\eta \lambda^{2}}\frac{1+\lambda a}{\Omega }$$

where

(116) $$E_{n}\left(\kappa a\right)={\left(\kappa a\right)}^{n-1}\int_{\kappa a}^{\infty }\frac{e^{-\kappa t}}{t^{n}}dt\;$$

is the exponential integral of order *n*.

Equation (115) is the required approximate expression for the electrophoretic mobility *μ* of a weakly charged oil drop with a slip length Λ in a charged polymer gel medium.

#### 2.4.2. Limiting Cases

Consider several limiting cases.

(i) In the limit of Λ ⟶ 0, Equation (115) reduces to

(117) $$\mu =\frac{{2\varepsilon }_{r}\varepsilon_{0}\zeta }{3\eta \Omega_{0}}\left[\frac{3\eta_{d}+3\eta +\eta \lambda a}{3\eta_{d}+2\eta }+\frac{\kappa \lambda a}{\kappa +\lambda }-\frac{\eta (1+\lambda a)(3+\kappa a)}{2(3\eta_{d}+2\eta )}+\frac{3\kappa^{2}}{2\lambda^{2}}\left\{1+\lambda a+\frac{\lambda^{2}a^{2}}{3}\left(\frac{3\eta_{d}+3\eta +\eta \lambda a}{3\eta_{d}+2\eta }\right)\right\}e^{\kappa a}E_{5}\left(\kappa a\right)\right.\;-\frac{3\kappa^{2}}{2\lambda^{2}}e^{\left(\kappa +\lambda \right)a}\left.\left\{E_{5}\left(\left(\kappa +\lambda \right)a\right)+\lambda aE_{4}\left(\left(\kappa +\lambda \right)a\right)+\frac{\lambda^{2}a^{2}}{3}E_{3}\left(\left(\kappa +\lambda \right)a\right)\right\}\right]-\frac{\rho_{fix}}{\eta \lambda^{2}}\frac{1+\lambda a}{\Omega_{0}}$$

with

(118) $$\Omega_{0}=1+\lambda a+\left(\frac{3\eta_{d}+3\eta +\eta \lambda a}{3\eta_{d}+2\eta }\right)\frac{\lambda^{2}a^{2}}{9}$$

Equation (117) agrees with the gel electrophoretic mobility *μ* of a weakly charged oil drop with a no-slip surface. In the further limit of $\rho_{fix}$ = 0, Equation (117) tends to

(119) $$\mu =\frac{{2\varepsilon }_{r}\varepsilon_{o}\zeta }{3\eta \Omega }\left[\frac{3\eta_{d}+3\eta +\eta \lambda a}{3\eta_{d}+2\eta }+\frac{\kappa \lambda a}{\kappa +\lambda }-\frac{\eta (1+\lambda a)(3+\kappa a)}{2(3\eta_{d}+2\eta )}+\frac{3\kappa^{2}}{2\lambda^{2}}\left\{1+\lambda a+\frac{\lambda^{2}a^{2}}{3}\left(\frac{3\eta_{d}+3\eta +\eta \lambda a}{3\eta_{d}+2\eta }\right)\right\}e^{\kappa a}E_{5}\left(\kappa a\right)\right.\;-\frac{3\kappa^{2}}{2\lambda^{2}}e^{\left(\kappa +\lambda \right)a}\left.\left\{E_{5}\left(\left(\kappa +\lambda \right)a\right)+\lambda aE_{4}\left(\left(\kappa +\lambda \right)a\right)+\frac{\lambda^{2}a^{2}}{3}E_{3}\left(\left(\kappa +\lambda \right)a\right)\right\}\right]$$

which agrees with the gel electrophoretic mobility *μ* of a weakly charged oil drop with a no-slip surface in an uncharged gel medium [59].

(ii) In the limit of *λa* ⟶ 0 and *ρ*_fix_ = 0, Equation (115) reduces to

(120) $$\mu =\frac{\varepsilon_{r}\varepsilon_{o}\zeta }{\eta }\left\{\frac{\kappa \Lambda a+\Lambda +a}{a+2\Lambda_{eff}}+2e^{\kappa a}E_{5}\left(\kappa a\right)-\frac{5a}{a+2\Lambda_{eff}}e^{\kappa a}E_{7}\left(\kappa a\right)\right\}$$

which agrees with the free-solution electrophoretic mobility *μ* of a weakly charged oil drop with a slip length Λ [57]. In the further limit of *η*_d_/*η* ⟶ ∞, Equation (120) tends to

(121) $$\mu =\frac{\varepsilon_{r}\varepsilon_{0}\zeta }{\eta }\left\{\frac{\kappa \Lambda a+\Lambda +a}{a+2\Lambda }+2e^{\kappa a}E_{5}\left(\kappa a\right)-\frac{5a}{a+2\Lambda }e^{\kappa a}E_{7}\left(\kappa a\right)\right\}$$

which agrees with the electrophoretic mobility of a rigid sphere with a slip length Λ in a free electrolyte solution [38]. In the further limit of Λ ⟶ 0, Equation (120) tends to the following Henry’s mobility expression of a rigid sphere with a no-slip surface [33]:

(122) $$\mu =\frac{\varepsilon_{r}\varepsilon_{o}\zeta }{\eta }\left\{1+2e^{\kappa a}E_{5}\left(\kappa a\right)-5e^{\kappa a}E_{7}\left(\kappa a\right)\right\}$$

(iii) In the limit of *κa* ⟶ ∞ (Smoluchowski limit), Equation (61) becomes

(123) $$\mu =\frac{\varepsilon_{r}\varepsilon_{0}\zeta (1+\lambda a)}{\eta \Omega }\left(\frac{\kappa \Lambda a+\Lambda +a}{a+2\Lambda_{eff}}\right)-\frac{\rho_{fix}}{\eta \lambda^{2}}\frac{1+\lambda a}{\Omega }\;$$

In the further limit of Λ ⟶ 0, Equation (123) tends to

(124) $$\mu =\frac{\varepsilon_{r}\varepsilon_{0}\zeta (1+\lambda a)}{\eta }\left(\frac{{3\eta }_{d}}{{3\eta }_{d}+2\eta }\right)-\frac{\rho_{fix}}{\eta \lambda^{2}}\left(1+\lambda a\;\right)$$

(iv) In the limit of *κa* ⟶ 0 (Hückel limit), Equation (115) tends to

(125) $$\mu =\frac{2\varepsilon_{r}\varepsilon_{0}\zeta }{3\eta \Omega \left(a+2\Lambda_{eff}\right)}\left\{a+\Lambda_{eff}\left(3+\lambda a\right)-\frac{\eta }{{2\eta }_{d}}\left(1+\lambda a\right)\right\}-\frac{\rho_{fix}}{\eta \lambda^{2}}\frac{1+\lambda a}{\Omega }\;$$

In the further limit of Λ = 0, Equation (125) becomes

(126) $$\mu =\frac{\varepsilon_{r}\varepsilon_{0}\zeta }{3\eta \Omega_{0}}\left\{\frac{{6\eta }_{d}+\eta \left(3-\lambda a\right)}{{3\eta }_{d}+2\eta }\right\}-\frac{\rho_{fix}}{\eta \lambda^{2}}\frac{1+\lambda a}{\Omega_{0}}\;$$

We now define the scaled gel electrophoretic mobility μ* as

(127) $$\mu =\frac{\varepsilon_{r}\varepsilon_{o}\zeta }{\eta }\mu^{*}$$

where

(128) $$\mu^{*}=\frac{2}{3\Omega }\left[\frac{a+\Lambda_{eff}\left(3+\lambda a\right)}{a+2\Lambda_{eff}}+\frac{\kappa \lambda a}{\kappa +\lambda }+\frac{a+\Lambda_{eff}\left(1+\lambda a\right)}{a+2\Lambda_{eff}}+\frac{3\kappa^{2}}{2\lambda^{2}}\left\{1+\lambda a+\frac{\lambda^{2}a^{2}}{3}\left(\frac{a+\Lambda_{eff}\left(3+\lambda a\right)}{a+2\Lambda_{eff}}\right)\right\}e^{\kappa a}E_{5}\left(\kappa a\right)\right.\;-\frac{3\kappa^{2}}{2\lambda^{2}}e^{\left(\kappa +\lambda \right)a}\left.\left\{E_{5}\left(\left(\kappa +\lambda \right)a\right)+\lambda aE_{4}\left(\left(\kappa +\lambda \right)a\right)+\frac{\lambda^{2}a^{2}}{3}E_{3}\left(\left(\kappa +\lambda \right)a\right)\right\}\right]-\frac{\eta a\left(1+\kappa a\right)\left(1+\lambda a\right)}{3\eta_{d}\Omega \left(a+2\Lambda_{eff}\right)}-\frac{\rho_{fix}}{\eta {\varepsilon_{r}\varepsilon_{o}\zeta \lambda }^{2}}\frac{1+\lambda a}{\Omega }$$

In the limit *λa* ⟶ 0, *η*_d_/*η* ⟶ ∞, and *ρ*_fix_ = 0, Equation (128) reduces to the Henry function of a spherical rigid particle in a free-electrolyte solution [33]:

(129) $$f\left(\kappa a\right)=1+2e^{\kappa a}E_{5}\left(\kappa a\right)-5e^{\kappa a}E_{7}\left(\kappa a\right)$$

#### 2.4.3. Dependence of the Electrophoretic Mobility on Various Parameters

**Figure 2:**
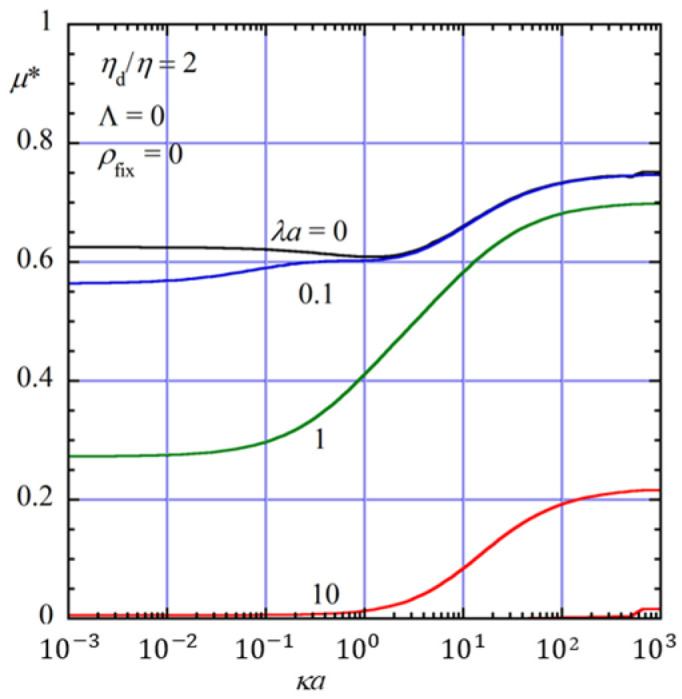
Scaled gel electrophoretic mobility μ* of an oil drop of radius *a* with a no-slip surface (Λ = 0) as a function of *κa* for four values of *λa* (0, 0,1, 1, 10) at *η*_d_/*η* = 2 in an uncharged gel (*ρ*_fix_ = 0). The curve with *λa* = 0 corresponds to the result for the free-solution electrophoresis.

Figure 2 shows the scaled gel electrophoretic mobility $\mu^{*}$ of an oil drop of viscosity *η*_d_ with a no-slip surface (Λ = 0) in an uncharged gel medium (*ρ*_fix_ = 0) of viscosity *η* and Brinkman screening length 1/*λ*, plotted as a function of *κa* for four values of *λa* (0, 0.1, 1, and 10) at *η*_d_/*η* = 2.

The parameter *λa* characterizes the strength of the hydrodynamic interaction between the oil drop and the polymer gel medium. The limiting case *λa* = 0 corresponds to the free-solution electrophoresis of an oil drop in the absence of the gel matrix.

As seen in Figure 2, the magnitude of the scaled mobility μ* decreases as *λa* increases over the entire range of *κa* considered. This behavior reflects the increasing hydrodynamic resistance exerted by the polymer gel network on the flow field around the drop. In other words, as *λa* becomes larger, the interaction between the oil drop and the surrounding gel medium becomes stronger, leading to a suppression of the electrophoretic motion of the drop. For sufficiently large values of λa, the mobility is significantly reduced compared with the free-solution case (*λa* = 0), demonstrating the important role of the gel matrix in modifying the electrophoretic behavior of the drop.

**Figure 3:**
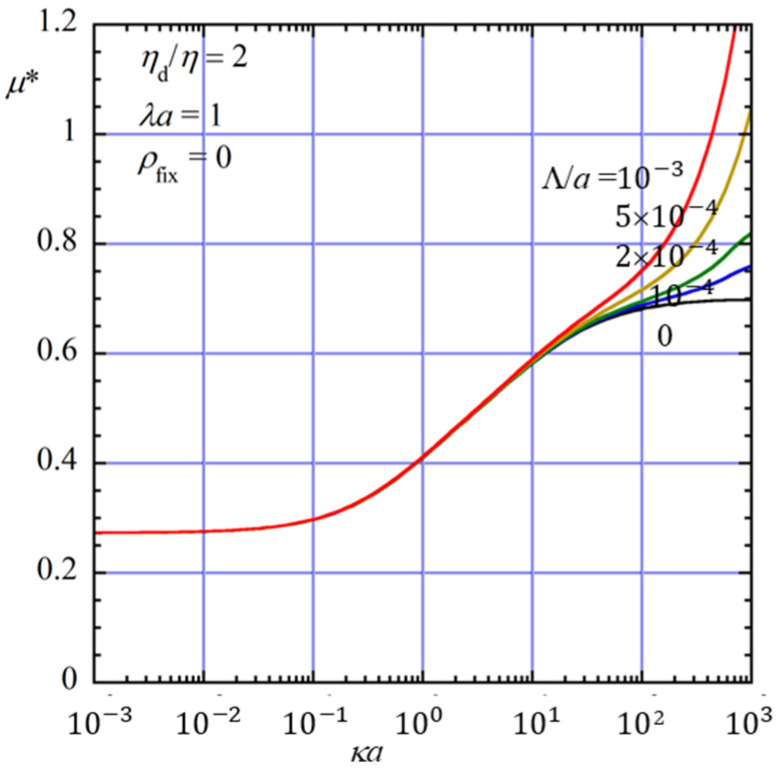
Scaled gel electrophoretic mobility μ* of an oil drop of radius *a* with a slip length Λ as a function of *κa* for five values of Λ/*a* (0, 10^−4^, 2 × 10^−4^, 5 × 10^−4^, and 10^−3^) at *η*_d_/*η* = 2 and *λa* = 1 in an uncharged gel (*ρ*_fix_ = 0). The curve with Λ/*a* = 0 corresponds to a drop with a no-slip surface.

Figure 3 shows the scaled gel electrophoretic mobility μ* as a function of *κa* for five values of the dimensionless slip length Λ/*a* (0, 10^−4^, 2 × 10^−4^, 5 × 10^−4^, and 10^−3^) at *η*_d_/*η* = 2 and *λa* = 1 in an uncharged gel medium (*ρ*_fix_ = 0). The case Λ/*a* = 0 corresponds to an oil drop with a no-slip surface.

As can be seen from Figure 3, the magnitude of the scaled mobility μ* increases as the dimensionless slip length Λ/*a* increases. However, this effect is negligible for small and moderate values of κa, and becomes pronounced only for sufficiently large *κa* (typically. *κa* ≳ 100). This behavior can be understood as follows. For large values of *κa*, the electric double layer is thin compared with the drop radius, and the drop surface locally behaves like a planar interface. In this limit, the influence of hydrodynamic slip at the drop surface becomes significant, because the reduction in tangential hydrodynamic resistance directly enhances the fluid motion near the interface. As a result, the increase in |μ*| with increasing Λ/*a* becomes more pronounced.

**Figure 4:**
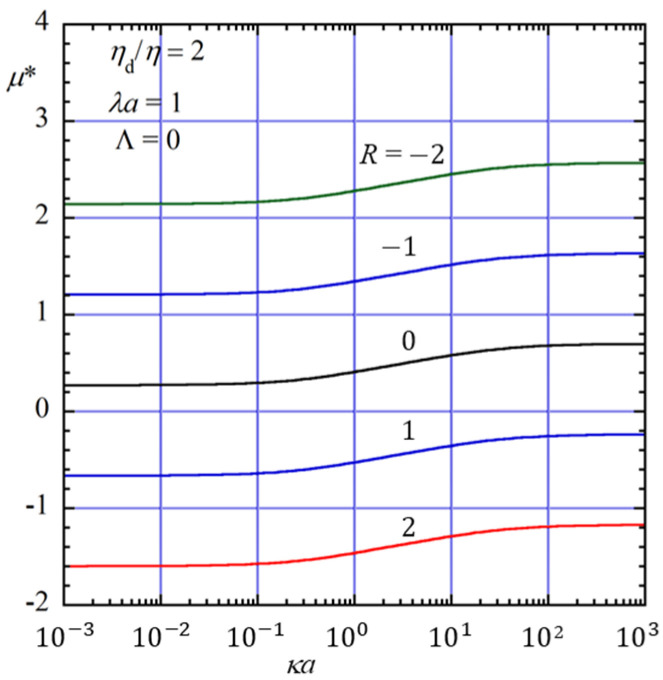
Scaled gel electrophoretic mobility μ* of an oil drop of radius *a* with a no-slip surface (Λ = 0) as a function of *κa* for five values of *R* (−2, −1, 0, 1, and 2) at *η*_d_/*η* = 2 and *λa* = 1 in an uncharged gel (*ρ*_fix_ = 0). The case *R* = 0 corresponds to an uncharged gel, for which no electroosmotic flow (EOF) occurs.

Figure 4 shows the dependence of the scaled gel electrophoretic mobility μ* on the contribution of the gel-fixed charge of density *ρ*_fix_, which induces electroosmotic flow (EOF) in the gel medium. To characterize the effect of the gel-fixed charge, we introduce the dimensionless parameter *R*, defined by

(130) $$R=\frac{a^{2}\rho_{fix}}{\varepsilon_{r}\varepsilon_{0}\zeta }$$

The parameter *R* represents the ratio of the gel-fixed volume charge density *ρ*_fix_ to the surface charge density *σ* of the oil drop. Note that *R* > 0 when *ρ*_fix_ and *σ* have the same sign, whereas *R* < 0 when they have opposite signs.

As seen in Figure 4, the scaled gel electrophoretic mobility μ* increases in magnitude for *R* < 0 and decreases for *R* > 0. This behavior arises from the electroosmotic flow generated by the gel-fixed charge. When *R* < 0, the electroosmotic flow is in the same direction as the electrophoretic motion of the drop, thereby enhancing the magnitude of the mobility. In contrast, when *R* > 0, the electroosmotic flow is in the opposite direction, leading to a reduction in the electrophoretic mobility. These results demonstrate that the presence of gel-fixed charges can significantly modify the electrophoretic behavior of the oil drop through the induced electroosmotic flow in the gel medium.

**Figure 5:**
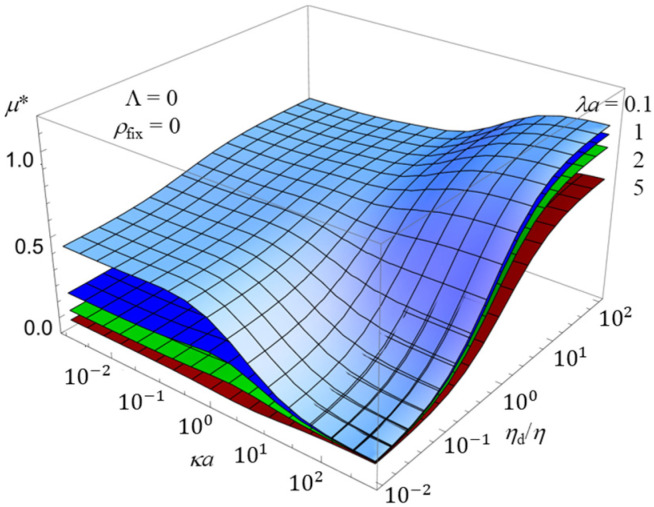
Three-dimensional plot of the scaled electrophoretic mobility μ* of an oil drop with a no-slip surface (Λ = 0) as a function of *κa* and *η*_d_/*η*, for four values of *λa* (0.1, 1, 2, and 5) in an uncharged gel (*ρ*_fix_ = 0).

**Figure 6:**
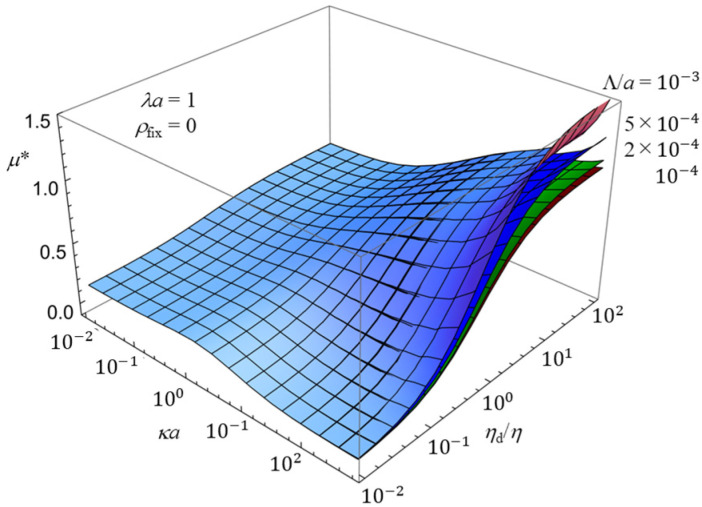
Three-dimensional plot of the scaled electrophoretic mobility μ* as a function of *κa* and *η*_d_/*η*, for five values of the scaled slip length Λ/*a* (10^−4^, 2 × 10^−4^, 5 × 10^−4^, and 10^−3^) in an uncharged gel (*ρ*_fix_ = 0).

To further elucidate the combined effects of multiple parameters on the electrophoretic mobility, three-dimensional plots are presented in Figure 5 and Figure 6. These figures complement the two-dimensional results shown in Figure 2, Figure 3 and Figure 4 by providing a more comprehensive view of the parameter dependence.

Figure 5 shows the scaled electrophoretic mobility μ* as a function of *κa* and the viscosity ratio *η*_d_/*η* for several values of the permeability parameter *λa* in the absence of hydrodynamic slip. It can be seen that the mobility depends sensitively on both parameters, and that their effects are not simply additive. In particular, the influence of the viscosity ratio becomes more pronounced at large *ka*, where the electric double layer is thin and interfacial hydrodynamics dominate the response.

Figure 6 presents the corresponding three-dimensional plot illustrating the effect of the scaled slip length Λ/*a*. The results show that the impact of hydrodynamic slip is strongly coupled with both *κa* and *η*_d_/*η*. In particular, the enhancement of the mobility due to slip becomes significant only in the regime of large κa, consistent with the trends observed in Figure 3. The three-dimensional representation clearly demonstrates how the interplay between interfacial slip and bulk hydrodynamic properties governs the overall electrophoretic behavior.

These results highlight the importance of considering multiple parameters simultaneously when analyzing electrophoresis in polymer gels, and demonstrate the usefulness of the present theoretical framework for capturing such coupled effects.

These results show that the gel electrophoretic mobility of an oil drop is strongly influenced by the hydrodynamic interaction with the polymer gel medium, the hydrodynamic slip at the drop surface, and the electroosmotic flow induced by gel-fixed charges. In particular, the mobility decreases with increasing gel resistance (larger λa), increases with increasing slip length Λ/a, and is either enhanced or reduced by the electroosmotic flow depending on the sign of the parameter R. These effects demonstrate the important roles of both hydrodynamic and electrokinetic interactions in determining the electrophoretic behavior of an oil drop in a polymer gel medium.

## 3. Conclusions

In this study, we have developed an analytical theory for the electrophoresis of weakly charged oil drops dispersed in a dilute polymer gel carrying fixed charges. By accounting for both the electrohydrodynamic response of the drop and the electroosmotic flow generated by the charged gel matrix, we derived a closed-form expression for the electrophoretic mobility in the low–zeta-potential regime (Equation (115)).

In contrast to uncharged gels, where the polymer network contributes only hydrodynamic resistance, a charged gel actively participates in electrokinetic transport through bulk electroosmosis. The measured mobility therefore reflects the superposition of two distinct mechanisms: the intrinsic electrophoretic motion of the drop and the background electroosmotic flow of the gel. The present formulation provides an explicit analytical representation of this coupling within a Brinkman–Debye–Bueche type porous-medium description [39,40].

Following the Baygents and Saville theory [43], the present theory assumes that the drop acquires charge through specific ion adsorption at the oil–water interface and neglects ion penetration into the drop interior. The analysis consistently incorporates interfacial tension gradients (Marangoni stresses) as well as hydrodynamic slip at the oil–water interface, which may be significant for hydrophobic drops in aqueous environments. These interfacial effects modify the stress balance at the drop surface and thereby influence the overall mobility.

The oil drop is assumed to be impermeable to ions, which is a reasonable approximation for typical oil–water systems where ions are not soluble in the oil phase. As a result, ionic transport is confined to the surrounding electrolyte solution, and the droplet interior does not contribute directly to charge transport.

In the present analysis, the drop is assumed to remain spherical. This assumption is justified when the interfacial tension is sufficiently large so that deformation due to electric stresses is negligible. Unlike soft particles, that is, polyelectrolyte-coated particles, where deformation can significantly affect electrophoretic mobility [61], oil droplets possess a well-defined interface that is impermeable to ions and fluid. As a result, deformation effects are generally less significant, although they may become important under conditions of low interfacial tension or strong electric fields.

Here, a linear isotherm is assumed for the surface charge, following the formulation of Baygents and Saville [42]. This corresponds to the limit of low surface coverage, where the surface charge density varies linearly with the local ionic concentration and saturation effects can be neglected. In more general situations, surface adsorption is described by nonlinear isotherms such as the Langmuir isotherm, and the present approximation is therefore expected to be valid primarily in the dilute or weak adsorption regime.

An important consequence of the derived mobility expression (Equation (115)) is that experimental measurements in charged gels can, in principle, be used to characterize both the drop and the gel. In particular, the formula provides a method for estimating the drop’s zeta potential and evaluating the electroosmotic mobility of the polymer network.

However, it should be noted that a single measurement of electrophoretic mobility under fixed conditions is generally not sufficient to uniquely determine both quantities, since different combinations of parameters may yield the same mobility. Reliable determination therefore requires measurements under varying conditions (e.g., electrolyte concentration or drop size) or additional independent information. The present theory thus provides a physically consistent and potentially useful framework for analyzing coupled electrophoresis–electroosmosis phenomena in charged polymer gels and related soft porous media.

## 4. Materials and Methods

The present work develops an analytical framework based on the fundamental equations describing electrokinetic transport in porous media. The treatment is restricted to the low–zeta-potential regime, allowing a linearized solution. The paper does not involve experimental procedures or computational simulations. All theoretical assumptions, governing equations, and derivations are presented in Section 2.

## Data Availability

Data are contained within the article.
